# First Complete Genome Sequence of a *Salmonella enterica* subsp. *enterica* Serovar Derby Strain Associated with Pork in France

**DOI:** 10.1128/genomeA.00853-15

**Published:** 2015-07-30

**Authors:** Annaëlle Kérouanton, Edouard Hirchaud, Valérie Rose, Emilie Esnault, Delphine Naquin, Martine Denis

**Affiliations:** a Anses, Hygiene and Quality of Poultry and Pig Products Unit, BP53, Site des Croix, Ploufragan, France; b Anses, Viral Genetics and Biosafety Unit, BP 53, Site des Croix, Ploufragan, France; c European University of Brittany, Rennes, France; d Institute for Integrative Biology of the Cell (I2BC), CEA, CNRS, Université Paris-Sud, Gif-sur-Yvette, France

## Abstract

In France, *Salmonella enterica* subsp. *enterica* serovar Derby is one of the most often isolated serovars in pigs. Here, we describe the draft genome sequence of a strain isolated from a pig. This strain had the most frequent pulsed-field gel electrophoresis (PFGE) and antimicrobial patterns (S, SSU, T) usually observed in pig production in France. Those patterns have been also highlighted in human isolates.

## GENOME ANNOUNCEMENT

*Salmonella enterica* subsp. *enterica* is the leading cause of bacterial foodborne disease in the world. *Salmonella* has the ability to colonize the guts of healthy pigs, which then can serve as carriers. When admitted to slaughterhouses, asymptomatic pigs become a potential risk for *Salmonella* contamination of pork meat and for *Salmonella* infections in humans. *S. enterica* subsp. *enterica* serovar Derby is, along with *S. enterica* subsp. *enterica* serovar Typhimurium, a major *Salmonella* serovar found in pigs in France and Europe (1). In humans, *S*. Derby usually ranks third or fourth in prevalence in France. Isolates of human and pig origins showed very high similarities in pulsed-field gel electrophoresis (PFGE) and in antimicrobial susceptibility (2). To assess the spread of isolates from pigs via pork to humans, PFGE proved to be highly useful and reliable, especially for tracking contamination sources (3, 4) and for outbreak investigations (5, 6). For *S*. Derby, however, the PFGE method after XbaI, BlnI, or SpeI restriction has shown low discriminatory power (7), so there is a need to have access to highly discriminatory typing methods based on sequence.

We present here the draft genome sequence of *S. enterica* subsp. *enterica* serovar Derby strain 07CR553 (Anses’ reference). This strain was isolated in 2007 from a pig carcass at a slaughterhouse and showed very common XbaI, BlnI, and SpeI PFGE patterns and antimicrobial patterns (S, SSU, T). Genomic DNA was isolated from overnight culture using the QIAamp DNA minikit (Qiagen, Courtaboeuf, France), followed by an RNase step.

For library preparation, genomic DNA samples were treated with a Covaris S220 focused ultrasonicator to obtain 350-bp DNA fragments (mean size). Ligation of Illumina TruSeq adaptors (DNA end-repair, dA-tailing, and ligation) were performed on an SPRI-TE instrument, using an SPRIworks fragment library system I kit (Beckman Coulter), according to the Illumina TruSeq DNA sample prep kit protocol. Libraries were amplified for 6 cycles using Kapa HiFi polymerase (Kapa Biosystems). Amplified libraries were size selected on an agarose gel (400 to 600 bp). Library quality was assessed on an Agilent Bioanalyzer 2100 using an Agilent high-sensitivity DNA kit.

Libraries were pooled in equimolar proportions and diluted to a final concentration of 10 pM, according to Illumina recommendations, and sequenced on an Illumina MiSeq instrument, using a MiSeq reagent V2 500 kit and a paired-end 2 × 250-bp recipe.

Reads were trimmed to 200 bp for R1 and 150 for R2 using PrinSeq (8). The Trinity normalize module was used for normalization (9). Genome assemblies were constructed *de novo* using Velvet version 5.1 (10) and generated 37 contigs and an *N*_50_ of 224 kb. The chromosome was found to be 4,826,293 bp, with a G+C content of 52%. A total of 4,509 potential coding sequences (CDSs) were highlighted. The CDSs were annotated using Prokka (11), and 7 rRNAs and 72 tRNAs were found.

This annotated complete genomic sequence is the first for a French *S*. Derby strain and will provide data for a better understanding of this serovar with highly conserved DNA.

### Nucleotide sequence accession numbers.

The complete genome for *S. enterica* subsp. *enterica* serovar Derby 07CR553 is available in GenBank under the accession no. LAZB00000000. The version described in this paper is the first version, LAZB01000000.
